# Impact of obstructive sleep apnea on prognosis of patients with cardiometabolic multimorbidity

**DOI:** 10.1186/s13098-024-01403-y

**Published:** 2024-07-26

**Authors:** Xiaogang Liu, Siyi Li, Xiao Wang, Jingyao Fan, Wei Gong, Yan Yan, Hui Ai, Bin Que, Wen Zheng, Shaoping Nie

**Affiliations:** 1 Center for Coronary Artery Disease, Division of Cardiology, Beijing Anzhen Hospital, Capital Medical University, No. 2 Anzhen Road, Chaoyang District, Beijing, 100029 China; 2 Beijing Institute of Heart, Lung, and Blood Vessel Diseases, Beijing, China; 3 Department of Cardiology, Wuhan Fourth Hospital, Wuhan, China

**Keywords:** Obstructive sleep apnea, Cardiometabolic multimorbidity, Acute coronary syndrome, Diabetes, Stroke

## Abstract

**Background:**

Cardiometabolic multimorbidity (CMM) is a growing global health problem, and obstructive sleep apnea (OSA) is recognized as an important risk factor for cardiovascular disease. However, the impact of OSA on the prognosis of CMM patients remains unclear.

**Methods:**

This study was a sub-study of OSA-acute coronary syndrome (ACS) and included 1, 927 hospitalized ACS patients. Patients were divided into the CMM group and the non-CMM group. OSA was diagnosed using the apnea-hypopnea index (AHI). The primary endpoint was major adverse cardiovascular and cerebrovascular events (MACCE). The secondary endpoint included cardiac events, all-cause death and all repeat revascularizations.

**Results:**

This study enrolled 1, 927 patients hospitalized for ACS, with a median follow-up of 3 years. Among them, 723 patients (37.5%) had CMM, while 1, 204 patients (62.5%) did not have CMM. Over half of the patients in each group had OSA. OSA patients exhibited worse cardiometabolic profiles than their non-OSA counterparts, including higher body mass index (BMI), glycemic indices, lipids and inflammation. In the CMM group, OSA patients had a significantly higher incidence of MACCE than non-OSA patients (34.7% vs. 23.7%, *p* = 0.004). These results remained significant after adjustment, indicating that OSA substantially increased the risk of MACCE in the CMM group (adjusted hazard ratio [HR]: 1.432; 95% confidence interval [CI]: 1.017–2.016; *p* = 0.040). Conversely, the incidence of MACCE was similar between OSA and non-OSA subgroups within the non-CMM cohort. Subgroup analyses showed that OSA increased the risk of MACCE in CMM patients aged ≥ 60 years (adjusted HR: 1.642; 95% CI: 1.031–2.615; *p* = 0.037) and in those with specific clinical characteristics.

**Conclusion:**

OSA significantly impacts the prognosis of CMM patients, highlighting the need for targeted OSA screening and management strategies to improve outcomes in this population potentially.

**Supplementary Information:**

The online version contains supplementary material available at 10.1186/s13098-024-01403-y.

## Introduction

As the global population ages, cardiometabolic multimorbidity (CMM) is increasingly prevalent and has emerged as a global health issue [1, 2]. In the United States, CMM rates among adults surged from 1999 to 2018, with a notable peak at 14.4% in 2017–2018 [3]. In Canada and South Asia, nearly one in four patients with cardiometabolic disease also suffered from CMM [4, 5]. CMM refers to the presence of at least two cardiometabolic diseases, such as acute coronary syndrome (ACS), diabetes, and stroke [6]. These diseases often interact and exacerbate each other, leading to a higher risk of overall mortality [7]. Despite its significant health impact, current research on CMM remains limited, and its contributing factors and prognosis are not well understood.

Obstructive sleep apnea (OSA) is a metabolic disorder linked to sleep disturbance, snoring, apnea, and fatigue [8]. This condition, which affects nearly a billion people globally, significantly raises the risk of cardiometabolic diseases and multi-organ damage [9]. OSA is prevalent among individuals with cardiometabolic diseases, affecting 46–66% of ACS patients and 49% of diabetic patients [10]. As a newly identified risk factor for cardiovascular diseases, OSA is closely related to the onset and progression of ACS, diabetes, stroke, and other cardiometabolic diseases, thereby increasing the risk of poor clinical outcomes [11–13]. However, current research primarily focuses on OSA’s impact on single cardiometabolic diseases, neglecting its influence on the prognosis of CMM.

Given the established link between OSA and cardiovascular disease, our study aims to explore the effect of OSA on the clinical prognosis of patients with CMM. This is the first comprehensive investigation into the impact of OSA on the prognosis of CMM patients. We hope that the findings from this study will contribute to effective strategies for improving prognosis and offer novel perspectives for future treatment approaches.

## Methods

### Study design and participants

This study is a subsection of the OSA-ACS project (NCT03362385), a single-center, large-scale, prospective cohort study conducted at Beijing Anzhen Hospital, Capital Medical University. The study population consisted of adult patients aged 18 to 85 years who were admitted with an ACS diagnosis from June 2015 to January 2020. Additionally, these patients were required to undergo an overnight sleep study. Exclusion criteria included cardiogenic shock/cardiac arrest, malignancy with a life expectancy of less than two years, and failed sleep studies. The study also excluded patients with less than 180 min of sleep, central sleep apnea, those receiving continuous positive airway pressure (CPAP), and those who were lost to follow-up. Patients were divided into the CMM group and the non-CMM group. CMM was defined as the coexistence of two or three cardiometabolic diseases, including ACS, diabetes, and stroke [14]. This study adhered to the STrengthening the Reporting of OBservational studies in Epidemiology (STROBE) guidelines [15] and complied with the revised Declaration of Helsinki [16].

### Overnight sleep monitoring

All patients underwent an overnight sleep study using a type III portable sleep monitoring device (ApneaLink Air, Resmed, Australia) once they were clinically stable. Trained researchers attached the sleep monitoring devices to the patients before bedtime, continuously monitored them throughout the night, and removed the devices the following morning. The devices recorded several signals, including nasal airflow, thoraco-abdominal movements, arterial oxygen saturation (SaO_2_), heart rate, and snoring episodes. The data was subsequently exported by investigators who were blinded to the patient’s baseline characteristics and stored in a specialized database for further analysis. According to apnea hyponea index (AHI), patients were divided into OSA (AHI ≥ 15 events·h^− 1^) and non-OSA (AHI < 15 events·h^− 1^) [17].

### Endpoints

The primary and secondary clinical endpoints were primarily identified through patient medical records, phone calls, and clinic visits conducted by researchers who were unaware of the patients’ basic profile and sleep monitoring results. All patients were followed for a minimum of six months post-discharge, with scheduled visits at 1 month, 3 months, 6 months, 12 months, and every 6 months thereafter. The primary endpoint was major adverse cardiovascular and cerebrovascular events (MACCE), including cardiovascular death, stroke, myocardial infarction, hospitalization for unstable angina or heart failure, and ischemia-driven revascularization. Secondary endpoints included cardiac events, all-cause death and all repeat revascularizations.

### Statistical analysis

Continuous variables were analyzed using descriptive statistics such as mean ± standard deviation (SD) and median (first and third quartiles), while categorical variables were analyzed using frequencies. The normality of continuous variables was assessed using the Shapiro-Wilks test. Appropriate statistical tests were used to compare groups. Kaplan-Meier curves were used to illustrate the cumulative incidences of primary and secondary endpoints, and the log-rank test was used for comparisons. Cox proportional hazards models were used to estimate the relationship between OSA and time to subsequent clinical events. All statistical analyses were performed using SPSS 26.0, with a two-sided p-value < 0.05 considered statistically significant.

## Results

### Patient characteristics

This prospective study included 1,927 patients hospitalized for ACS with a median follow-up of 3 years (Supplementary Fig. 1). Baseline CMM, defined as the coexistence of ≥ 2 of ACS, diabetes, and stroke, was present in 723 patients (37.5%). Within the CMM cohort, 383 patients (53.0%) had OSA based on sleep studies, while 340 patients (47.0%) did not. In the non-CMM group, 631 patients (52.4%) had OSA and 573 patients (47.6%) did not. In both groups, OSA patients had worse cardiometabolic profiles than their non-OSA counterparts, including higher body mass index (BMI) (OSA vs. non-OSA: 27.9 vs. 26.1 kg/m^2^ in CMM, 28.1 vs. 25.9 kg/m^2^ in non-CMM), glycemic indices (median hemoglobin A1c [HbA1c], OSA vs. non-OSA: 7.3% vs. 7.2% in CMM, 5.8% vs. 5.7% in non-CMM), lipids (median triglycerides, OSA vs. non-OSA: 1.6 vs. 1.4 mmol/L in CMM, 1.6 vs. 1.4 mmol/L in non-CMM) and inflammation (median high-sensitivity C-reactive protein [hs-CRP], OSA vs. non-OSA: 2.4 vs. 1.5 mg/L in CMM, 2.7 vs. 1.3 mg/L in non-CMM). In the non-CMM group, patients with OSA were predominantly male, had higher rates of hypertension and prior percutaneous coronary intervention (PCI), and were more likely to use ticagrelor and angiotensin-converting enzyme inhibitors/angiotensin receptor blockers (ACEIs/ARBs) (*p* < 0.05) (Table 1).

**Table 1:** Baseline characteristics of patients

Variables	CMM (*n* = 723)			Non-CMM (*n* = 1204)		
	OSA (*n* = 383)	Non-OSA (*n* = 340)	*P* value	OSA (*n* = 631)	Non-OSA (*n* = 573)	*P* value
Demographics						
Age, years	58.7 ± 10.1	58.1 ± 10.3	0.478	55.2 ± 10.6	55.1 ± 10.3	0.824
Male	314 (82.0)	266 (78.2)	0.207	572 (90.6)	477 (83.2)	< 0.001
BMI, kg/m^2^	27.9 ± 3.5	26.1 ± 3.3	< 0.001	28.1 ± 3.6	25.9 ± 3.5	< 0.001
**Medical History**						
Hypertension	295 (77.0)	244 (71.8)	0.105	396 (62.8)	312 (54.5)	0.003
Hyperlipidemia	164 (42.8)	128 (37.6)	0.157	179 (28.4)	166 (29.0)	0.817
Prior MI	76 (19.8)	54 (15.9)	0.166	101 (16.0)	85 (14.8)	0.574
Prior PCI	101 (26.4)	78 (22.9)	0.286	133 (21.1)	87 (15.2)	0.008
Prior CABG	11 (2.9)	8 (2.4)	0.663	7 (1.1)	3 (0.5)	0.423
Smoking			0.882			0.125
No	145 (37.9)	123 (36.2)		188 (29.8)	198 (34.6)	
Current	162 (42.3)	146 (42.9)		334 (52.9)	271 (47.3)	
Previous	76 (19.8)	71 (20.9)		109 (17.3)	104 (18.2)	
**Baseline Tests**						
Creatinine (µmol/l)	74.8 [65.2–87.3]	69.8 [61.0-80.6]	< 0.001	75.3 [65.7–84.3]	71.9 [63.8–81.7]	0.096
Hs-CRP, mg/L	2.4 [0.9–6.6]	1.5 [0.7–4.1]	0.001	2.7 [1.1–7.8]	1.3 [0.5–4.2]	< 0.001
eGFR, mL/min/1.73 m^2^	102.9 [83.4-117.2]	111.2 [90.9–126.0]	< 0.001	103.3 [89.8-120.4]	106.5 [92.3-123.5]	0.391
HbA1c (%)	7.3 [6.3–8.7]	7.2 [6.3–8.4]	0.167	5.8 [5.5–6.2]	5.7 [5.4–6.1]	0.001
HDL-c (mmol/L)	1.0 [0.8–1.1]	1.0 [0.8–1.1]	0.339	1.0 [0.9–1.2]	1.0 [0.9–1.2]	0.002
LDL-c (mmol/L)	2.4 [2.0–3.0]	2.3 [1.8–2.9]	0.238	2.6 [2.0-3.1]	2.5 [1.8–3.2]	0.499
Triglyceride (mmol/L)	1.6 [1.1–2.2]	1.4 [1.1–2.2]	0.121	1.6 [1.1–2.2]	1.4 [1.1–2.1]	0.019
TC (mmol/L)	4.0 [3.5–4.8]	3.9 [3.4–4.7]	0.399	4.3 [3.6-5.0]	4.2 [3.5-5.0]	0.758
LVEF, %	61.0 [55.0–65.0]	63.0 [58.0–66.0]	0.088	60.0 [55.8–65.0]	62.0 [57.0–66.0]	0.045
**Diagnosis**			0.102			0.157
STEMI	87 (22.7)	57 (16.8)		164 (26.0)	122 (21.3)	
NSTEMI	65 (17.0)	55 (16.2)		126 (20.0)	119 (20.8)	
Unstable angina	231 (60.3)	228 (67.1)		341 (54.0)	332 (57.9)	
**Procedures**						
Coronary angiography	374 (97.7)	329 (96.8)	0.469	616 (97.6)	558 (97.4)	0.789
PCI	239 (62.4)	199 (58.5)	0.288	428 (67.8)	343 (59.9)	0.004
CABG	30 (7.8)	32 (9.4)	0.449	29 (4.6)	39 (6.8)	0.097
Multivessel disease	275 (71.8)	238 (70.0)	0.594	400 (63.4)	332 (57.9)	0.053
Number of stents	1.0 [0.0–2.0]	1.0 [0.0–1.0]	0.338	1.0 [0.0–1.0]	1.0 [0.0–1.0]	0.023
**Medications on discharge**						
Aspirin	370 (96.6)	329 (96.8)	0.905	617 (97.8)	561 (97.9)	0.882
Clopidogrel	246 (64.2)	230 (67.6)	0.334	402 (63.7)	383 (66.8)	0.254
Ticagrelor	103 (26.9)	83 (24.4)	0.446	187 (29.6)	133 (23.2)	0.012
ACEIs/ARBs	256 (66.8)	213 (62.6)	0.238	409 (64.8)	317 (55.3)	0.001
β-blockers	306 (79.9)	257 (75.6)	0.164	493 (78.1)	432 (75.4)	0.261
Statins	376 (98.2)	333 (97.9)	0.822	621 (98.4)	567 (99.0)	0.416
**Sleep indicators**						
AHI, events·h^− 1^	28.4 [20.7–40.1]	7.5 [4.2–10.7]	< 0.001	28.7 [20.6–42.5]	7.9 [3.8–10.7]	< 0.001
ODI, events·h^− 1^	27.4 [20.8–37.8]	8.3 [5.0-11.7]	< 0.001	27.7 [20.0-39.9]	8.8 [4.7–12.0]	< 0.001
Minimum SaO_2_, %	82.0 [77.0–85.0]	87.0 [84.0–89.0]	< 0.001	83.0 [77.0–86.0]	88.0 [85.0–90.0]	< 0.001
Mean SaO_2_, %	93.0 [92.0–94.0]	94.0 [93.0–95.0]	< 0.001	93.0 [92.0–94.0]	94.0 [94.0–95.0]	< 0.001
Time with SaO_2_ < 90%, %	6.3 [2.0-16.6]	1.0 [0.1-3.0]	< 0.001	6.0 [2.0–15.0]	0.4 [0.0-2.1]	< 0.001
Epworth Sleepiness Scale	8.0 [5.0–12.0]	7.0 [3.0–11.0]	0.007	8.0 [4.0–12.0]	6.0 [3.0–10.0]	< 0.001

Data are given as mean ± SD, n (%) or median [IQR]. SD, standard deviation; IQR, interquartile range; CMM, cardiometabolic multimorbidity; OSA, obstructive sleep apnea; BMI, body mass index; MI, myocardial infarction; PCI, percutaneous coronary intervention; CABG, coronary artery bypass grafting; hs-CRP, high sensitivity C-reactive protein; eGFR, estimated glomerular filtration rate; HbA1c, hemoglobin A1C; HDL-c, high-density lipoprotein cholesterol; LDL-c, low-density lipoprotein cholesterol; TC, total cholesterol; LVEF, left ventricular ejection fraction; STEMI, ST segment elevation myocardial infarction; NSTEMI, non-ST segment elevation myocardial infarction; ACEIs, angiotensin converting enzyme inhibitors; ARBs, angiotensin receptor blockers; AHI, apnea hypopnea index; ODI, oxygen desaturation index; SaO_2_, arterial oxygen saturation

### Outcomes

**Fig. 1:**
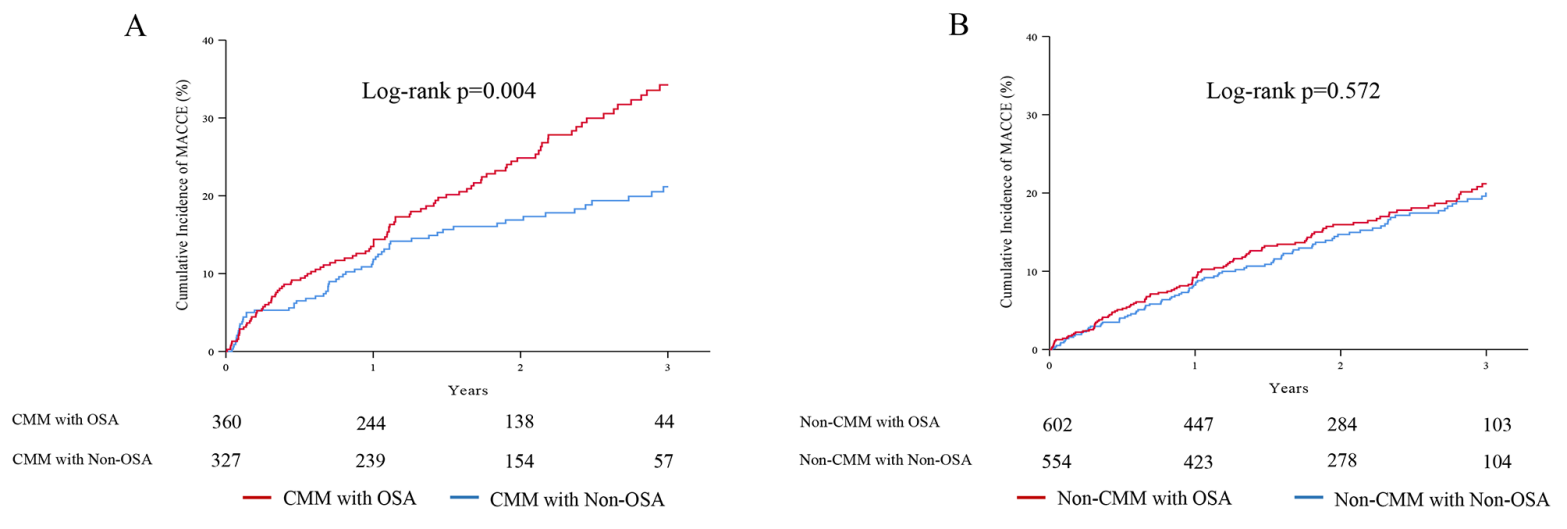
Kaplan-Meier analysis of cumulative incidence of MACCE in patients with CMM and the impact of OSA. **(A)** The effect of OSA on the cumulative incidence of MACCE in patients with CMM. **(B)** The effect of OSA on the cumulative incidence of MACCE in patients with non-CMM. MACCE, major adverse cardiovascular and cerebrovascular events; CMM, cardiometabolic multimorbidity; OSA, obstructive sleep apnea

During the 3-year follow-up, CMM patients with OSA had a significantly higher incidence of MACCE compared to those without OSA (34.7% vs. 23.7%, *p* = 0.004) (Table 2; Fig. 1A). These results remained significant after adjustment (adjusted hazard ratio [HR]: 1.432; 95% confidence interval [CI]: 1.017–2.016; *p* = 0.040), indicating that OSA significantly increased the risk of MACCE in CMM patients (Table 3). Conversely, over the 3-year follow-up, the incidence of MACCE was similar between the OSA and non-OSA subgroups within the non-CMM cohort (22.7% vs. 21.3%; *p* = 0.572) (Table 2; Fig. 1B). After multivariate adjustment, the difference remained non-significant (adjusted HR: 1.013; 95% CI: 0.745–1.378; *p* = 0.934) (Table 3).

**Table 2:** Three-year clinical outcomes in patients with CMM stratified by OSA Status

Variables	CMM (*n* = 723)			Non-CMM (*n* = 1204)		
	OSA (*n* = 383)	Non-OSA (*n* = 340)	*P* value	OSA (*n* = 631)	Non-OSA (*n* = 573)	*P* value
**MACCE**	99 (34.7)	61 (23.7)	0.004	107 (22.7)	93 (21.3)	0.572
Cardiovascular death	9 (3.4)	8 (3.3)	0.950	7 (1.6)	5 (1.2)	0.651
Myocardial infarction	10 (3.7)	5 (2.1)	0.231	19 (4.2)	10 (2.4)	0.129
Stroke	16 (5.9)	12 (4.9)	0.546	6 (1.3)	6 (1.4)	0.919
Hospitalization for unstable angina	63 (22.7)	41 (16.3)	0.049	79 (17.0)	71 (16.4)	0.761
Hospitalization for heart failure	7 (2.6)	3 (1.2)	0.240	2 (0.4)	4 (1.0)	0.384
Ischemia-driven revascularization	34 (12.5)	26 (10.4)	0.444	50 (10.8)	36 (8.5)	0.210
**Cardiac events§**	85 (30.3)	53 (20.9)	0.011	102 (21.7)	87 (20.0)	0.485
**All-cause death**	12 (4.5)	14 (5.7)	0.564	8 (1.8)	8 (1.9)	0.883
**All repeat revascularization**	49 (17.7)	34 (13.5)	0.175	70 (15.0)	59 (13.6)	0.561
PCI	46 (16.6)	33 (13.1)	0.247	65 (14.0)	53 (12.3)	0.445
CABG	3 (1.1)	1 (0.4)	0.342	5 (1.1)	6 (1.4)	0.655
Target vessel revascularization	24 (8.8)	20 (8.1)	0.711	34 (7.4)	27 (6.4)	0.507
Non-target vessel revascularization	35 (12.7)	16 (6.5)	0.015	46 (10.0)	37 (8.7)	0.527

Data are given as n (%). § Include cardiovascular death, myocardial infarction, ischemia-driven revascularization, or hospitalization for unstable angina or heart failure. CMM, cardiometabolic multimorbidity; OSA, obstructive sleep apnea; MACCE, major adverse cardiovascular and cerebrovascular events; PCI, percutaneous coronary intervention; CABG, coronary artery bypass grafting

**Table 3:** Univariable and Multivariable Cox Models to assess the Effect of OSA on primary and secondary outcomes

Variables	CMM (*n* = 723)				Non-CMM (*n* = 1204)			
	Unadjusted	*P* value	Adjusted	*P* value	Unadjusted	*P* value	Adjusted	*P* value
**MACCE**	1.594 [1.158–2.194]	0.004	1.432 [1.017–2.016]	0.040	1.083 [0.821–1.431]	0.572	1.013 [0.745–1.378]	0.934
Cardiovascular death	1.031 [0.398–2.672]	0.950	0.943 [0.348–2.556]	0.908	1.302 [0.413–4.103]	0.652	0.999 [0.296–3.365]	0.998
Myocardial infarction	1.907 [0.651–5.579]	0.239	1.253 [0.398–3.947]	0.700	1.796 [0.835–3.862]	0.134	1.695 [0.718-4.000]	0.228
Stroke	1.259 [0.595–2.661]	0.547	1.024 [0.458–2.286]	0.955	0.943 [0.304–2.924]	0.919	0.860 [0.243–3.041]	0.814
Hospitalization for unstable angina	1.480 [0.999–2.194]	0.051	1.435 [0.943–2.186]	0.092	1.051 [0.763–1.448]	0.761	1.046 [0.734–1.492]	0.802
Hospitalization for heart failure	2.203 [0.570–8.523]	0.252	1.222 [0.288–5.181]	0.786	0.478 [0.088–2.612]	0.395	0.372 [0.061–2.285]	0.286
Ischemia-driven revascularization	1.221 [0.732–2.034]	0.445	1.105 [0.635–1.924]	0.724	1.314 [0.856–2.017]	0.212	1.327 [0.823–2.140]	0.246
**Cardiac events§**	1.560 [1.106–2.199]	0.011	1.412 [0.978–2.039]	0.066	1.107 [0.832–1.474]	0.485	1.038 [0.756–1.426]	0.816
**All-cause death**	0.797 [0.369–1.724]	0.565	0.874 [0.378–2.018]	0.752	0.929 [0.349–2.476]	0.883	0.649 [0.221–1.911]	0.433
**All repeat revascularization**	1.352 [0.873–2.905]	0.176	1.261 [0.786–2.023]	0.336	1.108 [0.784–1.567]	0.562	1.034 [0.705–1.518]	0.864
PCI	1.301 [0.832–2.035]	0.249	1.220 [0.751–1.982]	0.422	1.152 [0.801–1.656]	0.445	1.085 [0.726–1.622]	0.690
CABG	2.853 [0.297–27.438]	0.364	3.159 [0.235–42.407]	0.385	0.763 [0.233–2.502]	0.656	0.661 [0.172–2.547]	0.548
Target vessel revascularization	1.119 [0.618–2.026]	0.711	1.073 [0.561–2.054]	0.831	1.187 [0.716–1.967]	0.507	1.321 [0.759–2.297]	0.325
Non-target vessel revascularization	2.054 [1.137–3.712]	0.017	1.993 [1.069–3.715]	0.030	1.150 [0.746–1.773]	0.528	1.014 [0.625–1.646]	0.955

Adjusted Cox models for age, male, BMI, hypertension, hyperlipidemia, smoking, hs-CRP, eGFR. § Include cardiovascular death, myocardial infarction, ischemia-driven revascularization, or hospitalization for unstable angina or heart failure. OSA, obstructive sleep apnea; CMM, cardiometabolic multimorbidity; MACCE, major adverse cardiovascular and cerebrovascular events; PCI, percutaneous coronary intervention; CABG, coronary artery bypass grafting; BMI, body mass index; hs-CRP, high sensitivity C-reactive protein; eGFR, estimated glomerular filtration rate

In CMM patients, OSA was also linked to a more than 40% increase in hospitalization for unstable angina compared to the non-OSA group (22.7% vs. 16.3%, *p* = 0.049; adjusted HR: 1.435; 95% CI: 0.943–2.186; *p* = 0.092). Composite cardiac events were also increased in CMM-OSA patients (30.3% vs. 20.9%, *p* = 0.011; adjusted HR: 1.412; 95% CI: 0.978–2.039; *p* = 0.066). Furthermore, the higher rate of non-target vessel revascularization in the CMM-OSA group (12.7% vs. 6.5%, *p* = 0.015; adjusted HR: 1.993; 95% CI: 1.069–3.715; *p* = 0.030) suggests that OSA may accelerate de novo atherosclerotic changes. Again, there were no differences in outcomes between the OSA and non-OSA groups in non-CMM patients during the 3-year follow-up (Tables 2 and 3).

### Subgroup analysis

We performed subgroup analyses based on age, sex, BMI, hypertension, hyperlipidemia, myocardial infarction, diagnosis, and PCI/coronary artery bypass grafting (CABG) (Table 4). The association between the presence of OSA and increased risk of MACCE was strongest in CMM patients with age ≥ 60 years (adjusted HR: 1.642; 95% CI: 1.031–2.615; *p* = 0.037), BMI < 28 kg/m^2^ (adjusted HR: 1.708; 95% CI: 1.108–2.633; *p* = 0.015), hyperlipidemia (adjusted HR: 1.947; 95% CI: 1. 064-3.563; *p* = 0.031) and prior myocardial infarction (adjusted HR: 3.786; 95% CI: 1.478–9.693; *p* = 0.006), suggesting that the adverse cardiovascular effects of OSA were more pronounced in those with a greater degree of metabolic dysfunction or preexisting vascular damage. In the subgroups of non-ST segment elevation ACS (NSTE-ACS) and those not treated with PCI/CABG, the presence of OSA had a more pronounced effect on MACCE in CMM patients, and this difference remained statistically significant after adjustment for confounders.

**Table 4:** Subgroup Analysis of OSA’s effect on MACCE assessed by Multivariable Cox Models

Variables	CMM (*n* = 723)		Non-CMM (*n* = 1204)	
	Adjusted	*P* value	Adjusted	*P* value
Age (years)*				
<60	1.288 [0.776–2.137]	0.327	1.288 [0.854–1.945]	0.228
≥60	1.642 [1.031–2.615]	0.037	0.857 [0.533–1.378]	0.524
Gender*				
male	1.385 [0.941–2.037]	0.099	0.943 [0.676–1.317]	0.732
female	1.961 [0.911–4.222]	0.085	1.661 [0.709–3.889]	0.243
BMI*				
<28	1.708 [1.108–2.633]	0.015	1.064 [0.730–1.550]	0.746
≥28	0.995 [0.593–1.670]	0.986	0.813 [0.487–1.356]	0.427
Hypertension*				
Yes	1.455 [0.988–2.144]	0.058	1.145 [0.773–1.698]	0.499
No	1.448 [0.690–3.037]	0.328	0.827 [0.498–1.375]	0.464
Hyperlipidemia*				
Yes	1.947 [1.064–3.563]	0.031	0.984 [0.523–1.851]	0.959
No	1.213 [0.792–1.857]	0.374	1.050 [0.735-1.500]	0.788
Myocardial infarction				
Yes	3.786 [1.478–9.693]	0.006	1.166 [0.563–2.418]	0.679
No	1.166 [0.797–1.707]	0.428	0.971 [0.690–1.367]	0.865
Diagnosis				
STEMI	1.357 [0.681–2.704]	0.386	1.144 [0.621–2.105]	0.666
NSTE-ACS	1.501 [1.007–2.239]	0.046	0.933 [0.652–1.337]	0.707
PCI/CABG				
Yes	1.223 [0.804–1.859]	0.346	0.877 [0.616–1.247]	0.464
No	2.150 [1.155–4.002]	0.016	1.492 [0.783–2.843]	0.224

*There was no adjustment for gender, age, BMI, hypertension, or hyperlipidemia. Adjusted Cox models for age, male, BMI, hypertension, hyperlipidemia, smoking, hs-CRP, eGFR. OSA, obstructive sleep apnea; CMM, cardiometabolic multimorbidity; MACCE, major adverse cardiovascular and cerebrovascular events; BMI, body mass index; STEMI, ST segment elevation myocardial infarction; NSTE-ACS, non-ST segment elevation-acute coronary syndrome; PCI, percutaneous coronary intervention; CABG, coronary artery bypass grafting; hs-CRP, high sensitivity C-reactive protein; eGFR, estimated glomerular filtration rate

## Discussion

In this prospective cohort study, we observed a strong association between OSA and CMM. OSA significantly elevated the risk of MACCE in CMM patients, and this association remained significant even after adjusting for confounders using a multivariate Cox regression model over a 3-year follow-up period. However, the impact of OSA on prognosis in the non-CMM group was not significant. This study is the first to investigate the effect of OSA on adverse cardiovascular outcomes in CMM, highlighting the necessity for enhanced OSA screening in this patient population.

Previous research has extensively studied OSA’s impact on individual cardiovascular or metabolic diseases. OSA has been identified as an emerging risk factor for cardiovascular diseases and associated complications [8, 13, 18–20]. OSA alone has been shown to increase the risk of coronary events, presumably due to repeated episodes of hypoxemia and reoxygenation, which result in systemic inflammation and oxidative stress [21]. Moreover, OSA significantly increased the risk of diabetic macrovascular and microvascular complications, playing a significant role in various cardiometabolic diseases [11, 22]. Numerous studies have demonstrated a significant association between insulin resistance markers, such as the triglyceride-glucose index (TyG index) and various lipid indicators, and OSA. In a meta-analysis that included 10 studies involving 16,726 participants, the severity of OSA was significantly associated with the TyG index. The TyG index in OSA patients was notably higher than that in healthy controls (standardized mean difference [SMD] = 0.856, 95% CI = 0.579–1.132, *p* < 0.001), indicating a higher risk of insulin resistance among these patients [23]. Furthermore, a systematic review and meta-analysis by *Abud et al.* indicated that CPAP therapy could partially improve metabolic disturbances and insulin resistance in OSA patients [24]. Besides the TyG index, other lipid indicators such as triglycerides, total cholesterol, high-density lipoprotein (HDL), and low-density lipoprotein (LDL) are also associated with OSA [25]. Research has pointed out that OSA patients often exhibit abnormal lipid metabolism, including elevated levels of triglycerides, total cholesterol, and LDL, and reduced levels of HDL [26]. These lipid abnormalities may further contribute to the development of insulin resistance. Moreover, some newer lipid indices have been found to be related to OSA, with the most significant being the visceral adiposity index (VAI), atherogenic index of plasma (AIP), and lipid accumulation product (LAP). In a meta-analysis that included 14 original studies involving 14,943 patients, it was found that OSA patients had significantly higher levels of VAI, AIP, and LAP, which increased with the severity of OSA [27]. A meta-analysis by *Cattazzo et al.*, which included 31 randomized controlled trials (RCTs), demonstrated that CPAP therapy could improve total cholesterol levels in OSA patients, but it did not significantly reduce triglycerides, HDL, and LDL levels [28]. Therefore, there is a clear link between OSA and metabolic disorders, highlighting the importance of managing OSA to improve metabolic health.

CMM, a complex chronic health condition, has a more substantial negative impact on adult life expectancy worldwide compared to a single cardiometabolic disease [29, 30]. CMM refers to the coexistence of two or more cardiometabolic diseases and represents one of the most common and severe forms of multimorbidity [31]. Compared with non-CMM patients, those with CMM demonstrated a higher cumulative incidence of MACCE. Our study is the first to demonstrate that OSA does not increase the risk of MACCE in non-CMM patients but significantly impacts the clinical outcomes of CMM patients. These findings underscore the importance of OSA screening in this population.

Our subgroup analysis aligns with prior studies, demonstrating that age is a critical factor in MACCE risk for OSA patients with cardiovascular disease [32]. The findings underscore the complex interactions between OSA, CMM, and individual factors, advocating for personalized treatment strategies and emphasizing the need for further research. In particular, the role of hypoxia in MACCE risk warrants deeper exploration, as it remains underexamined in the current literature.

In addition, our study findings hold significant clinical utility and application value for primary care physicians. Given the global increase in aging populations, more patients are presenting with multiple CMM, and primary care physicians are the first point of contact for diagnosis and health management in these cases. Our research indicated that OSA significantly impacted adverse outcomes in patients with CMM. This suggests that screening and treating OSA in such populations is crucial for improving their prognoses. However, despite OSA being a novel risk factor for cardiovascular diseases [33], many patients are aware of their symptoms but do not take the condition seriously and do not seek appropriate testing and treatment [34]. This phenomenon is especially prevalent in developing countries. Therefore, as the frontline force in primary healthcare, primary care physicians should be vigilant about the presence of OSA symptoms in patients with CMM. If symptoms are present, further screening to confirm the diagnosis is warranted. Additionally, physicians should provide health education to these patients, informing them about OSA and its implications, and offer lifestyle recommendations and follow-up treatments such as CPAP [35]. These measures could potentially reduce the risk of MACCE in patients with CMM.

OSA synergistically increases the risk of adverse outcomes in patients with CMM. The risk of MACCE is higher in OSA patients with CMM. However, we did not find significant differences in individual adverse outcomes between the OSA and non-OSA groups. This may be due to the need for a larger sample size. The statistical significance observed in MACCE is likely attributed to the cumulative effect of various adverse cardiovascular and cerebrovascular events. Future studies should consider expanding the sample size for validation.

### Strengths and Limitations

Our study has several strengths. Firstly, our study is based on the largest prospective OSA-ACS cohort in China, with a substantial sample size, ensuring the reliability and credibility of our findings. Secondly, the study focuses on OSA as an emerging risk factor for cardiovascular diseases, providing the first in-depth exploration of its clinical prognosis impact in CMM patients. This underscores the importance of OSA screening in this population. Lastly, our cohort study has a long follow-up duration with ongoing follow-up, providing crucial reference for the long-term prognosis of OSA in CMM patients, which holds significant clinical relevance given the current global trend of aging populations with multiple coexisting diseases.

This study has the following limitations. Firstly, this study was conducted at a single center, which may introduce bias in the sample selection. Secondly, the diagnosis of OSA based on portable polygraphy, which may underestimate the severity of OSA. However, it can still serve as an alternative to polysomnography. Thirdly, the severity of OSA was likely to be overestimated during the acute phase of ACS. However, sleep monitoring was usually conducted during the clinically stable period after admission (average of two days), and other literature supported this assessment of OSA in acute phases, including heart failure.

## Conclusion

This study revealed a strong association between OSA and poor outcomes in patients with CMM. Specifically, OSA significantly increased the incidence of adverse events in CMM patients. This was not observed in non-CMM patients, suggesting that the effects of OSA were more pronounced in multimorbidity. In subgroups, OSA significantly increased the risk of MACCE, particularly in older adults (≥ 60 years), patients with hyperlipidemia, myocardial infarction, NSTE-ACS, and patients not treated with PCI/CABG. In summary, our findings highlight the importance of OSA in the prognosis of CMM, emphasizing the need for targeted OSA screening and management strategies to improve outcomes in this population potentially.

### Electronic supplementary material

Below is the link to the electronic supplementary material.

Supplementary Material 1

## Data Availability

No datasets were generated or analysed during the current study.
