# Supplementary material for: Impact of obstructive sleep apnea on prognosis of patients with cardiometabolic multimorbidity
Source: Diabetol Metab Syndr. 2024 Jul 26;16:178. doi: 10.1186/s13098-024-01403-y (PMC11282628; doi:10.1186/s13098-024-01403-y)
Supplement: Supplementary file 1 — Supplementary Material 1 [file 13098_2024_1403_MOESM1_ESM.docx]

**Supplemental Figure 1.** Flowchart of the study. CPAP, continuous positive airway pressure; CMM, cardiometabolic multimorbidity; OSA, obstructive sleep apnea.
